# The efficacy of Na-butyrate encapsulated in palm fat on performance of broilers infected with necrotic enteritis with gene expression analysis

**DOI:** 10.14202/vetworld.2016.450-457

**Published:** 2016-05-09

**Authors:** M. G. Eshak, M. A. Elmenawey, A. Atta, H. B. Gharib, B. Shalaby, M. H. H. Awaad

**Affiliations:** 1 Department of Cell Biology, National Research Centre, Dokki, Cairo, Egypt; 2 Department of Animal Production, Faculty of Agriculture, Cairo University, Cairo, Egypt; 3 Department of Bacteriology, Animal Health Research Institute, Dokki, Cairo, Egypt; 4 Department of Poultry Diseases, Faculty of Veterinary Medicine, Cairo University, Cairo, Egypt

**Keywords:** chickens, deoxyribonucleic acid fragmentation, gene expression analysis, Na-butyrate, necrotic enteritis

## Abstract

**Aim:**

To study the efficacy of Na-butyrate encapsulated in palm fat on performance of broiler chickens experimentally infected with necrotic enteritis (NE) with the determination of its protective effect against the changes in the gene expression profiles and deoxyribonucleic acid (DNA) fragmentation.

**Materials and Methods:**

A total of 800 one-day-old male Arbor Acres Plus broiler chickens were randomly allocated into four groups for 5 weeks. Na-butyrate was supplemented at dosages of 1 kg/ton for starter diet, 0.5 kg/ton for grower diet, and 0.25 kg/ton for finisher diet (presence or absence). Birds of groups 1 and 2 were inoculated by crop gavages with 4×10^8^ CFU/ml/bird of *Clostridium perfringens* in phosphate buffered saline for 4 successive days, from 14 to 17 days of age to produce NE.

**Results:**

Addition of Na-butyrate, encapsulated in palm fat, to ration of experimentally infected broilers with NE resulted in increased final body weight, at 35 days of age, reduced total feed consumption, improved feed conversion ratio, reduced cumulative mortality, and increased production number. There were increased intestinal diameter, intestinal length, and significantly increased the weight of bursa of Fabricius(BF) with higher hemagglutination inhibition titers against Newcastle disease (ND) vaccination versus untreated infected and untreated negative control birds. The results showed increased expression levels of alpha-toxin and glyceraldehyde-3-phosphate dehydrogenase in the bursa tissues of broilers infected with *C. perfringens*. However, the expression levels of these genes in broilers treated with Na-butyrate were similar to the non-infected control group. Supplementation of broilers with Na-butyrate increased the expression level of insulin-like growth factor-1 (IGF-1) and decreased the DNA fragmentation induced by *C. perfringens*.

**Conclusion:**

Na-butyrate significantly improved chicken broiler body weights, increased relative weights of BF, increased antibody titers against ND vaccination, numerically lowered mortality due to *C. perfringens* infection, increased the expression level of IGF-1, and decreased the DNA fragmentation induced by *C. perfringens*. Obtained results point out the effectiveness of Na-butyrate encapsulated in palm fat in improving the production performance variables, immune response, and intestinal morphology in experimentally induced NE as well as in non-infected chicken broilers.

## Introduction

It is already established that chicken gastrointestinal tract (GIT) provides a means by which the body derives nutrition, furnishes protective mechanisms to safeguard the host, and serves as an environment for other living organisms [1]. *Clostridium perfringens* belongs to the resident microbiota [2]; however, this microorganism along with predisposing factors such as mucosal damage is prerequisites for developing of necrotic enteritis (NE) [3]. Many recent studies of NE have focused on finding different ways to control this disease [4].

Butyrate is of special interest due to its numerous positive effects on the health of gut and extraintestinal tissues. Butyrate is the most important energy source of the colonocytes [5]. It regulates the proliferation and differentiation of the gastrointestinal epithelium [6] and induces apoptosis in genetically disordered cells [7]. As a consequence, butyrate has a protective effect against colorectal cancer, which was reported in some *in vitro* [8] and also *in vivo* animal studies [9]. Due to its selective antimicrobial action on most enteric pathogens [10], butyrate improves the balance of the intestinal microflora which can influence the health of the host animal or the human host [11].

Accordingly, this study was dedicated to determine the effect of using Na-butyrate encapsulated in palm fat (which is a protected acidifier encapsulated in a vegetable fat matrix, resulting in a slow release of the acids during transport through the intestinal tract) on the productive performance variables of experimentally induced NE in chicken broilers with determination of its protective effect against the changes in the gene expression profiles and deoxyribonucleic acid (DNA) fragmentation.

## Materials and Methods

### Ethical approval

The experiment was carried out according to the National regulations on animal welfare and approved by the Institutional Animal Ethical Committee. This study was carried out at the Poultry Research Center, Department of Animal Production, Faculty of Agriculture, Cairo University, Giza, Egypt.

### Experimental birds

One-day-old male Arbor Acres Plus broiler chickens (n=800) were assigned at random to four equal experimental groups (1-4) of 200 birds assigned into 10 replicates of deep litter pens (2*1 m) with 20 birds per replicate. The brooding temperature was set at 32°C on the first day, gradually reduced to 24°C by the end of the third week, and kept at that level until the end of the experiment. The lighting pattern was 23 h L:1 h D. All chicks were vaccinated against ND at the 7^th^ and 21^st^ day of age using live Hitchner B1 and La Sota strain vaccines, respectively. Live infectious bursal disease vaccine (IBD 228-E vaccine) was administrated at the 14^th^ day of age. Drinking water method was used as a route of administration of the live vaccines. On the 10^th^ day of age, 0.5 ml inactivated avian influenza vaccine (H5N1) was injected subcutaneously in the back of neck [12]. Broilers were housed in the semi-closed house. The composition of the diets and their calculated analysis are shown in Table-1. The diets used were formulated to meet the nutrient requirements of the broiler chicks during starter, grower, and finisher periods according to the National Research Council [13]. Semduramicin was added to all rations at a concentration of 25 ppm as a coccidiostat. No antibiotics were administrated in water or feed, for the whole experimental period (35 days). Birds had free access to feed and water.

**Table-1:** Composition of the broilers 3-phase diets (g/kg as fed) and their calculated chemical composition.

Corn gluten meal 60%	70	70	66.5
Soya oil	30	43.8	40
Di-calcium phosphate	18	18	18
Lime stone	13	13	13
D.L. Methionine	2.2	2.1	2.3
Lysine hydrochloride	2.9	2.8	3.6
Sodium chloride	4	4	4
Premix*	3	3	3
Calculated analysis			
Crude protein %	23.0	21.0	19.0
Metabolizable energy (kcal/kg)	3000	3100	3200

* Each 3 g of premix contained: Vitamin A (trans-retinyl acetate), 9,000 IU

Vitamin D3 (cholecalciferol), 2,600 IU; Vitamin E (dl-á-tocopheryl acetate), 16 mg; Vitamin B1, 1.6 mg; Vitamin B2, 6.5 mg; Vitamin B6, 2.2 mg; Vitamin B12 (cyanocobalamin), 0.015 mg; Vitamin K3, 2.5 mg; choline (choline chloride), 300 mg; nicotinic acid, 30 mg; pantothenic acid (d-calcium pantothenate), 10 mg; folic acid, 0.6 mg; d-biotin, 0.07 mg; manganese (MnO), 70 mg; zinc (ZnO), 60 mg; iron (FeSO H O), 40 mg; copper (CuSO 5H O), 7 mg; iodine [Ca(IO)], mg; selenium (NaSeO), 0.3 mg

### Experimental design

Birds of groups 1 and 3 received Na-butyrate in palm fat (which is fat coated sodium of alimentary fatty acid). Its ingredient is n-Butyric acid sodium salt 30±2%. Na-butyrate supplementation in ration was given at a dosages according to the manufacturer’s recommendations (starter diet: 1 kg/ton, grower diet: 0.5 kg/ton, and finisher diet: 0.25 kg/ton). Birds of groups 2 and 4 received control ration without treatment. Birds of groups 1 and 2 were inoculated by crop gavages with 4×10^8^ CFU/ml/bird of *C. perfringens* in phosphate buffered saline (PBS) for 4 successive days, from 14 to 17 days of age [14]. The used strain of *C. perfringens* was type A B2 NET B, isolated from cases of chicken NE. Broilers of groups 3 and 4 were kept without infection.

### Measured parameters

#### Productive performance

Chicken performance response variables were determined according to North [15]; weekly individual body weight (wt.) was measured on all birds. Weekly feed consumption (g/d/bird), feed conversion ratio (FCR) (g feed/g live body wt. gain), and mortality rate were measured for each replicate. Dead birds were weighed to include their weights in the feed conversion estimates. An index of productivity is the so-called production number, which equals (Kilograms of growth per day * (100-mortality%)/Feed conversion ratio)*100 [16] was estimated for each replicate, at the end of the experimental period.

#### Intestinal length and diameter

Intestinal length (duodenum + jejunum + ileum) and diameter (in the middle of ileum) were measured on three birds from each replicate (chosen at random), on the 35^th^ day of age.

#### Relative weights of spleen, thymus, and BF

At 35 days of age, determination of the relative spleen, thymus, and BF weights, as a percent of the fasting live body weights, was performed on the chosen 3 birds from each replicate.

#### Humoral anti-ND vaccine antibody titers

For determination of the effect of NE infection and feed supplementation with Na-butyrate on humoral immunity, blood samples were collected from wing veins of 30 randomly selected birds from each replicate, at 2, 3, 4, and 5 weeks of age. Serum samples were subjected to HI test for determining antibody titers against ND vaccination as described by Swayne *et al*. [17].

The present study was also aimed to: (1) Investigate the role the immune proteins encoding genes (alpha-toxin and glyceraldehyde-3-phosphate dehydrogenase [GPD]) in bursa tissues of broilers treated with *C. perfringens*, (2) Examine the alteration in insulin-like growth factor-1 (IGF-1) gene expression due to *C. perfringens* exposure in liver tissues, (3) Study the effect of *C. perfringens* on the DNA fragmentation in the intestinal tissues of treated broilers, and (4) Evaluate the protective effect of Na-butyrate against the changes in the gene expression and DNA fragmentation induced by *C. perfringens* in broiler organs.

#### DNA fragmentation in intestine using gel electrophoresis laddering assay

Apoptotic DNA fragmentation of 10 birds from each treatment (one from each replicate), at 5 weeks of age, was qualitatively analyzed by detecting the laddering pattern of nuclear DNA according to Gibb *et al*. [18]. Briefly, intestinal tissues were homogenized, washed in PBS, and lysed in 0.5 ml of DNA extraction buffer (50 mM Tris–HCl, 10 mM EDTA. 0.5% Triton, and 100 μg/ml proteinase K, pH 8.0) for overnight at 37°C. The lysate was then incubated with 100 μg/ml, DNase-free, RNase for 2 h at 37°C, followed by three extractions of an equal volume of phenol/chloroform (1:1 v/v) and a subsequent re-extraction with chloroform by centrifuging at 15,000 rpm for 5 min at 4°C. The extracted DNA was precipitated in two volumes of ice-cold 100% ethanol with 1/10 volume of 3 M sodium acetate, pH 5.2 at −20°C for 1 h, followed by centrifuging at 15,000 rpm for 15 min at 4°C. After washing with 70% ethanol, the DNA pellet was air-dried and dissolved in 10 mM Tris–HCl/1 mM EDTA, pH 8.0. The DNA was then electrophoresed on 1.5% agarose gel and stained with ethidium bromide in Tris/acetate/EDTA (TAE) buffer (pH 8.5, 2 mM EDTA, and 40 mM Tris–acetate). A 100-bp DNA ladder (Invitrogen, USA) was included as a molecular size marker, and DNA fragments were visualized and photographed by exposing the gels to ultraviolet trans illumination.

### Gene expression analysis

Extraction of total RNA: The bursa and liver tissues of 10 birds from each treatment (one from each replicate), at 5 weeks of age, were used individually to extract total RNA using TRIzol^®^ Reagent (Invitrogen, Germany). Total RNA of each tissue was treated individually with 1 U of RQ1, RNAse-free, DNAse (Invitrogen, Germany) to digest DNA residues, re-suspended in DEPC-treated water, and photospectrometrically quantified at A260. The purity of total RNA was assessed by the 260/280 nm ratio (between 1.8 and 2.1). In addition, integrity was assured with ethidium bromide-stain analysis of 28S and 18S bands by formaldehyde-containing agarose gel electrophoresis. Aliquots were used immediately for reverse transcription (RT), otherwise stored at −80°C.

### Synthesis of the cDNA using RT reaction

The complete Poly (A)^+^ RNA isolated from birds tissues was reverse transcribed into cDNA in a total volume of 20 µl using Revert Aid^™^ First Strand cDNA Synthesis Kit (MBI Fermentas, Germany). An amount of total RNA (5µg) was used as a reaction mixture, termed as master mix (MM). The MM was consisted of 50 mM MgCl_2_,5× RT buffer (50 mM KCl; 10 mM Tris-HCl; pH 8.3), 10 mM of each dNTP, 50 µM oligo-dT primer, 20 U ribonuclease inhibitor (50 kDa recombinant enzyme to inhibit RNase activity), and 50 U M-MuLV reverse transcriptase. The mixture of each sample was centrifuged for 30 s at 1000 g and transferred to the thermocycler (Biometra GmbH, Göttingen, Germany). The RT reaction was carried out at 25°C for 10 min followed by 1 h at 42°C and finished with a denaturation step at 99°C for 5 min [19,20]. Afterward, the reaction tubes containing RT preparations were flash-cooled in an ice chamber until being used for DNA amplification through semi-quantitative real time-polymerase chain reaction (sqRT-PCR).

### sqRT-PCR

PCR reactions were set up in 25 µL reaction mixtures containing 12.5 µL 1× SYBR^®^ Premix Ex TaqTM (TaKaRa, Biotech. Co. Ltd.), 0.5 µL 0.2 µM sense primer, 0.5 µL 0.2 µM antisense primer, 6.5 µL distilled water, and 5 µL of cDNA template. The reaction program was allocated to 3 steps. The first step was at 95.0°C for 3 min. The second step consisted of 40 cycles in which each cycle divided into 3 steps: (a) At 95.0°C for 15 s; (b) at 55.0°C for 30 s; (c) at 72.0°C for 30 s. The third step consisted of 71 cycles which started at 60.0°C and then increased about 0.5°C every 10 s up to 95.0°C. At the end of each sqRT-PCR, a melting curve analysis was performed at 95.0°C to check the quality of the used primers. Each experiment included a distilled water control. The quantitative values of RT-PCR of Alpha-toxin (Forward: CCG CTC GAG TTG GGA TGG AAA AAT TGA T; Reverse: CCG GAA TTC TTT ATA TTA TAA GTT GAA TTT [21], GPD (Forward: CCG CTC GAG GGT AAA AGT AGC TAT TAA CGG; Reverse: CCG GGT ACC TTA GAA ACT AAG CAT TTT AAA [21] and IGF-1 (Forward: GCT GTT TCC TGT CTA CAG TG; Reverse: GTA CTC TGC AGA TGG CAC AT, GenBank accession no. M32791) genes were normalized on the bases of β-actin (β-actin-F): 5’-TGT GAT GGT GGG AAT GGG TCA G-3’, B-actin-R: 5’-TTT GAT GTCACG CAC GAT TTC C -3′, expression [22]. At the end of each sqRT-PCR, a melting curve analysis was performed at 95.0°C to check the quality of the used primers.

### Calculation of gene expression

The amplification efficiency (Ef) was calculated from the slope of the standard curve using the following formula [23]: Ef=10^−1/slope^ Efficiency (%) = (Ef−1) × 100. The relative quantification of the target to the reference was determined using the ΔC_T_ method if E for the target (Alpha-toxin, GPD, and IGF-1), and the reference primers (β-Actin) are the same [24]. Ratio_(reference/target gene)_=Ef^C^_T_^(reference)^−^C(target)^ Gene expression data are expressed as means±standard error of mean.

### Statistical analyzes

One-way analysis of variance has been adopted using SAS software general linear models procedure [25]. Percentage data were subjected to arcsine transformation before analysis. Mean values were compared using Duncan’s multiple range test [26] when significant differences existed. Significance was set at p<0.05.

## Results

### Production performance

The results revealed that NE infected birds consumed the basal diet had significantly the lowest body weights, at 35 days of age versus the other three groups. However, non-infected birds consumed diet with Na-butyrate had significantly the heaviest body weights. The other two treatment groups (control birds and infected birds consumed Na-butyrate) body weights were intermediate with no significant differences between them. Data of feed consumption and FCR indicated that there were no significant effects due to either *C. perfringens* infection or Na-butyrate supplementation (Table-2). Numerical higher mortality rate was present in infected consumed basal diet group as compared with the other three groups. Supplementation of Na-butyrate to non-infected birds decreased the total mortality percentage. Our data indicated that the other two experimental groups had intermediate mortalities with no significant differences between them (Table-2).

**Table-2:** Effect of Na-butyrate feed supplementation on performance and mortality of CPI and non-infected broilers.

Body weight (g)	1 d	1 weeks	2 weeks	3 weeks	4 weeks	5 weeks
Na-butyrate+CPI	45.3±0.1	173.4±0.7	486.3±2.1^ab^**	960.7±6.4^a^	1427±7.2^b^	1852±8.8^b^
CPI	45.1±0.2	174.0±0.6	482.1±3.6^b^	938.1±6.5^b^	1398±7.6^c^	1822±9.9^c^
Na-butyrate	44.7±0.2	175.1±0.6	492.9±2.9^a^	963.1±4.7^a^	1480±7.1^a^	1915±7.3^a^
Negative control	44.9±0.2	173.0±0.9	484.6±2.6^b^	942.9±5.1^b^	1460±7.1^a^	1863±9.7^b^
Probability	0.0881	0.1887	0.0439	0.0027	0.0001	0.0001

**Feed consumption** **(g/bird/day)**	**1 week**	**2 weeks**	**3 weeks**	**4 weeks**	**5 weeks**	**Cumulative (g/bird)**

Na-butyrate+CPI	22.7±0.48	52.1±0.94	109.6±2.32	125.7±2.53	160.9±2.68	3291±34.90
CPI	23.9±0.44	54.3±0.52	104.7±1.68	123.0±3.94	165.4±7.39	3298±78.45
Na-butyrate	23.6±0.27	54.4±0.44	106.4±1.86	127.1±2.23	163.7±2.18	3326±34.57
Negative control	23.5±0.41	54.1±0.75	105.4±1.18	122.4±2.79	165.7±3.52	3296±44.42
Probability	0.1961	0.0765	0.2432	0.6379	0.8677	0.9692

**Feed conversion** **(g feed/g live body weightgain)**	**1 week**	**2 weeks**	**3 weeks**	**4 weeks**	**5 weeks**	

Na-butyrate+CPI	0.918±0.025	1.067±0.025	1.339±0.026	1.518±0.018	1.779±0.023	
CPI	0.962±0.015	1.137±0.021	1.366±0.019	1.531±0.025	1.810±0.043	
Na-butyrate	0.929±0.007	1.108±0.013	1.341±0.019	1.474±0.019	1.737±0.018	
Negative control	0.956±0.017	1.121±0.023	1.364±0.025	1.462±0.021	1.770±0.028	
Probability	0.2209	0.1253	0.7488	0.0714	0.3987	

**Weekly mortality rate (%)**	**1 week**	**2 weeks**	**3 weeks**	**4 weeks**	**5 weeks**	**Cumulative mortality rate**

Na-butyrate+CPI	2.22±1.13	2.59±0.96	0.00±0.00	1.48±0.82	2.59±1.24	8.88±1.93
CPI	2.59±0.79	1.48±0.82	1.11±1.11	3.33±1.51	4.07±1.78	12.58±3.27
Na-butyrate	2.96±0.49	1.48±0.60	0.37±0.37	1.48±0.82	1.11±0.57	7.40±1.23
Negative control	2.22±0.82	1.85±0.83	1.11±0.57	1.85±0.99	3.70±1.10	10.73±1.60
Probability	0.9104	0.7441	0.5410	0.5727	0.3493	0.3650

** Means with different superscripts are significantly different (p≤0.05). CPI=*Clostridium perfringens* infected

NE infection resulted in a significant decrease in the production number as compared to non-infected birds that consumed Na-butyrate diet (Table-3). However, there were no significant differences between these two groups and the other groups. However, the lowest production number was that of the infected group while the highest production number was that of the non-infected Na-butyrate-treated group.

**Table-3:** Effect of Na-butyrate feed supplementation on production number of CPI and non-infected broilers.

Treatment	Production number
Na-butyrate+CPI	264.4±6.06^ab^**
CPI	245.3±14.25^b^
Na-butyrate	285.0±6.90^a^
Negative control	262.0±8.28^ab^
Probability	0.0059

** Means with different superscripts are significantly different (p≤0.05). CPI=*Clostridium perfringens* infected

### Intestinal length and diameter

The birds infected with NE had significantly shorter intestine as compared to the non-infected ones that consumed Na-butyrate diet and the control group. However, there were no significant differences between infected birds that consumed Na-butyrate diet and control group. There were no significant effects of either NE infection or Na-butyrate supplementation on the intestinal diameter (Table-4).

**Table-4:** Effect of Na-butyrate feed supplementation on relative weights of thymus, spleen and bursa of Fabriciusand intestinal length and diameter of CPI and non-infected broilers.

Treatment	Trait (%)				

	Thymus	Spleen	Bursa	Intestinal length (cm)	Intestinal diameter (cm)
Na-butyrate+CPI	0.780±0.04	0.205±0.005	0.265±0.024^a^*	196.5±1.82^bc^	0.960±0.018
CPI	0.700±0.02	0.210±0.007	0.210±0.007^b^	194.5±1.49^c^	0.955±0.021
Na-butyrate	0.740±0.03	0.220±0.009	0.220±0.009^b^	201.3±1.45^a^	0.990±0.010
Negative control	0.745±0.03	0.200±0.007	0.200±0.009^b^	199.5±1.02^ab^	0.985±0.015
Probability	0.3059	0.1430	0.0056	0.0083	0.3491

** Means with different superscripts are significantly different (p≤0.05). CPI=*Clostridium perfringens* infected

### Relative weights of spleen, thymus, and bursa of Fabricius

There were no significant effects of either NE infection or Na-butyrate supplementation on the relative weights of thymus or spleen, for 35-day-old male broilers (Table-4). On the other hand, birds that were infected with NE and consumed Na-butyrate diet had significantly the highest relative weights of the bursa of Fabricius, as compared to the other three groups.

### Humoral anti-ND vaccine antibody titers

Serum antibody responses to vaccination against ND antigen that were determined at 2, 3, 4, and 5 weeks of age are presented in Table-5. NE infection significantly lowered HI titers against ND as compared to other groups at all studied intervals. NE infected, or non-infected birds that consumed Na-butyrate diet had significantly higher antibody titers, as compared to the groups that consumed basal ration.

**Table-5:** Effect of Na-butyrate feed supplementation on weekly antibody titers against Newcastle virus vaccine of CPI and non-infected broilers.

Treatment	HI Titer			

	2 weeks	3 weeks	4 weeks	5 weeks
Na-butyrate+CPI	6.20±0.41^a^*	5.70±0.27^a^	5.10±0.33^a^	6.80±0.25^a^
CPI	4.00±0.59^b^	4.10±0.16^b^	3.30±0.21^b^	3.50±0.28^c^
Na-butyrate	6.20±0.27^a^	5.90±0.16^a^	6.00±0.25^a^	7.20±0.20^a^
Negative control	5.10±0.42^ab^	5.80±0.25^a^	5.88±0.52^a^	5.44±0.38^b^
Probability	0.0012	0.0001	0.0001	0.0001

* Means with different, superscripts, within age are significantly different (p≤0.05). CPI=*Clostridium perfringens* infected

### DNA fragmentation using gel electrophoresis laddering assay

**Figure-1:**
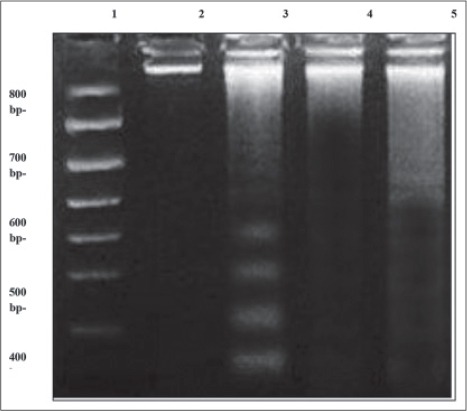
DNA fragmentation detected with agarose gel of DNA extracted from intestinal tissues of chicken by DNA gel electrophoresis laddering assay. Lane 1 represents DNA ladder. Lane 2 represents negative control chickens. Lane 3 represents chickens infected with *C. perfringens*. Lane 4 represents chickens treated with Na-butyrate. Lane 5 represents chickens infected with *C. perfringens* and treated with Na-butyrate.

The results of gel electrophoresis laddering assay showed that the supplementation with NA-butyrate resulted in very low DNA damage which was relatively similar to the negative control birds (Figure-1). Where, the DNA bands resulted from the damaged DNA were very low in the supplemented birds with Na-butyrate. However, NE infected birds expressed more DNA bands compared with the control ones or those supplemented with Na-butyrate. On the contrary, the damaged DNA due to *C. perfringens* infection from birds consumed Na-butyrate had decreased DNA fragmentation versus infected birds with *C. perfringens* alone (Figure-1).

**Figure-2:**
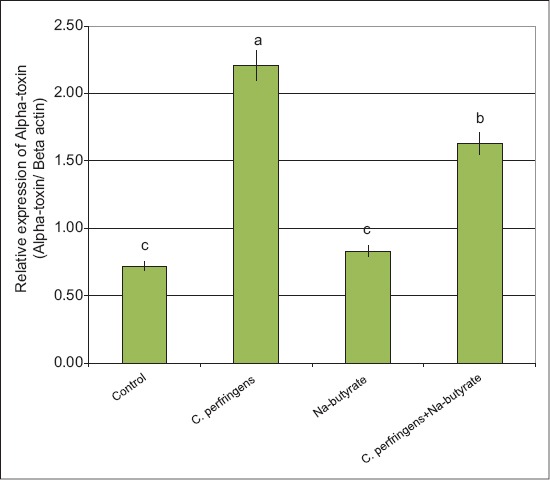
Semi-quantitative real time-polymerase chain reaction analysis of alpha-toxin-mRNAs in bursa tissues collected from chickens infected with *C. perfringens* and/or Na-butyrate. ^a,b,c^Means with different letters, differ significantly (p≤0.05).

**Figure-3:**
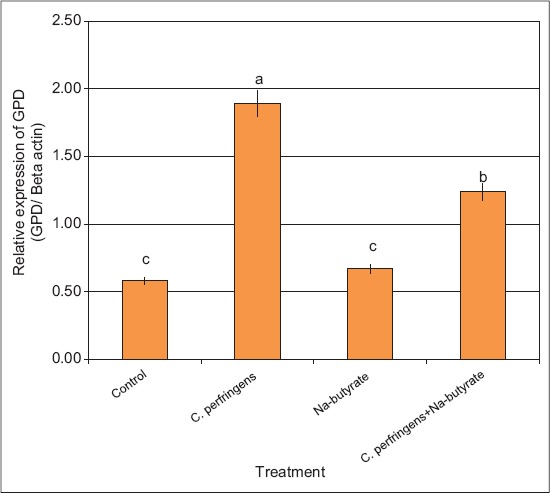
Semi-quantitative real time-polymerase chain reaction analysis of GPD-mRNAs in bursa tissues collected from chickens infected with *C. perfringens* and/or Na-butyrate. ^a,b,c^Means with different letters, differ significantly (p≤0.05).

**Figure-4:**
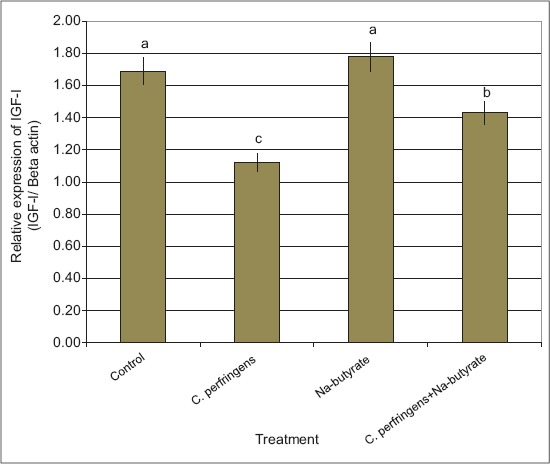
Semi-quantitative real time-polymerase chain reaction analysis of insulin-like growth factor-1 - mRNAs in liver tissues collected from chickens infected with *C. perfringens* and/or Na-butyrate. ^a,b,c^Means with different letters, differ significantly (p≤0.05).

Expression of immune protein encoding genes (alpha-toxin and GPD) and IGF-1 gene: The results revealed that the expression levels of alpha-toxin and GPD genes were significantly higher in birds infected with *C. perfringens* versus any of the other groups (Figures-2 and 3). However, the expression levels of alpha-toxin and GPD genes in birds supplemented with Na-butyrate were similar to the control birds. In addition, *C. perfringens* infection and supplementation with Na-butyrate showed expression levels of alpha-toxin and GPD genes significantly lower than the infected birds with *C. perfringens* alone; however, it was still significantly higher than the negative control group (Figures-2 and 3). The expression level of IGF-1 gene decreased significantly in NE infected birds as compared with that in the control ones (Figure-4). However, the expression level of the IGF-1 gene in birds supplemented with Na-butyrate was significantly higher than that in infected birds and relatively similar to that of the control group. In addition, the infection with *C. perfringens* and supplementation with Na-butyrate significantly increased the expression level of IGF-1 compared with that in birds infected with *C. perfringens* alone (Figure-4) provided that it was still significantly lower than the negative control group.

## Discussion

Na-butyrate supplementation to the ration of non-infected broiler chickens for 35 days resulted in increased final body weight (52.4 g), decreased feed consumption (30 g), improved FCR (33 points), lowered cumulative mortality (3.33%) with increased production number (23.0), increased intestinal diameter (0.005 cm), and intestinal length (2 cm) and increased BF weight (2%) with higher HI antibody titers against ND vaccine as compared with their untreated negative controls. Addition of similar dose of Na-butyrate to the ration of NE infected birds resulted in increased final body weight (29.2 g), decreased feed consumption (6.5 g), improved FCR (31 points), lowered cumulative mortality (3.7%) with increased production number (19.1), increased intestinal diameter (0.005 cm), and intestinal length (1.8 cm) with significant increase in BF weight (5.5%) and higher HI antibody titers against ND vaccine, as compared with the infected untreated group. Recently, several clinical studies indicated that butyric acid or its sodium salt mediated the immunity response [27]. The obtained results might attributed to the fact that organic acids have properties of lowering the intestinal pH, enhancing protein digestion, influencing intestinal cell morphology, stimulating pancreatic secretions, acting as a substrate for the intermediary metabolism, improving the retention of many nutrients (e.g. chelating minerals), increase intestinal integrity and influencing the electrolyte balance in the feed and intestine [28,29]. Taking in consideration that many countries have banned or limited the general use of antibiotics in feed as growth promoters (AGP) in animals [8]; the positive effect of Na-butyrate encapsulated in palm fat on performance variables could be used alternatively to AGP which confirms the results of Awaad *et al*. [30], who reported that usage of protected organic acids, in poultry nutrition, could be an efficacious tool to replace AGP. Moreover, Gauthier [29] concluded that organic acids could be a powerful tool in maintaining the health of poultry GIT, thus improving their production performances. Jankowskia *et al*. [31] reported that the use of butyric acid in poultry nutrition is quite well accepted due to the reduction of pH that limits the development of pathogens and helps in the digestion of proteins. They explained its mode of action as: Once Na-butyrate reaches the stomach of the bird, it quickly release sodium ion and, due to the low pH, butyrate is rapidly converted to the undissociated form, termed the butyric acid. This form is the one responsible for the antimicrobial activity, as the butyric acid is strongly lipophilic and can diffuse across the membranes of bacteria.

Experimental induction of NE in broiler chickens in the present investigation increased expression levels of alpha-toxin and GPD genes in the bursa tissues. Kulkarni *et al*. [21] found that expression of alpha-toxin and GPD genes were highly expressed in broilers infected with *C. perfringens*. On the other hand, the expression levels of these genes in broilers supplemented with Na-butyrate in the present work were similar to the negative control group. Moreover, treatment of broilers with Na-butyrate increased the expression level of IGF-1 and decreased the DNA fragmentation induced by NE infection. These results are in agreement with those reported by Jankowskia *et al*. [31]. Recent studies have shown that the effects of butyrate in the intestinal lumen are induced via the activation of specific receptors in the epithelial cells. When present in the lower part of the intestinal tract, butyrate can eliminate colonization of detrimental bacteria inhibiting the expression of the gene that is responsible for the invasion of the epithelial cells [21]. These previous findings could give us an explanation that why Na-butyrate enhanced the growth and inhibited the DNA fragmentation in the intestine (due to the butyric acid role which suppresses the infection that induced DNA damage).

## Conclusion

The present investigation proved the effectiveness of Na-butyrate encapsulated in palm fat in improving the production performance variables, immune response, and intestinal morphology in experimentally induced NE as well as in non-infected chicken broilers. Na-butyrate supplementation alleviated the negative effects of NE infection on broiler alpha-toxin and GPD genes in the bursa tissues and increased the expression level of IGF-1 and decreased intestinal DNA fragmentation induced by NE infection.

## Authors’ Contributions

MGE carried out the DNA fragmentation and gene expression analyzes. A. Atta applied the immune status assessment. HBG and ME carried out the productive performance together with the statistical analysis of the obtained results. BS carried out the bacteriological work. MHHA planed the investigation. All authors participated in draft and revision of the manuscript. All authors read and approved the final manuscript.

## References

[1] Aarestrup F.M (1999). Association between the consumption of antimicrobial agents in animal husbandry and the occurrence of resistant bacteria among food animals. Int. J. Antimicrob. Agends.

[2] Sengupta N, Alam S, Kumar R, Singh L (2011). Diversity and antibiotic susceptibility pattern of cultivable anaerobic bacteria from soil and sewage samples of India. Infect. Genet. Evol.

[3] Llanco L.A, Nakano V, Ferreiranda A, Avila-Campos M (2012). Toxinotyping and antimicrobial susceptibility of *Clostridium perfringens* isolated from broiler chickens with necrotic enteritis. Int. J. Microbiol. Res.

[4] Shojadoost B, Andrew R, John F (2012). The successful experimental induction of necrotic enteritis in chickens by *Clostridium perfringens* a critical review. Vet. Res.

[5] Roediger W.E (1982). Utilization of nutrients by isolated epithelial cells of the rat colon. Gastroenterology.

[6] Gá lfi P, Neográ dy S (2002). The pH-dependent inhibitory action of n-butyrate on gastrointestinal epithelial cell division. Food Res. Int.

[7] Leu R, Hu Y, Brown I, Young G (2009). Effect of high amylase maize starches on colonic fermentation and apoptotic response to DNA-damage in the colon of rats. Nutr. Metab.

[8] Young G.P, Gibson P.R (1995). Butyrate and the human cancer cell.

[9] LeLeu R, Brown I, Hu Y, Morita T, Esterman A, Young G (2007). Effect of dietary resistant starch and protein on colonic fermentation and intestinal tumourigenesis in rats. Carcinogenesis.

[10] Ferná ndez-Rubio C, Ordó nez C, Abad-Gonzá lez J, Garcia-Gallego A, Pilar Honrubia M, Jose Mallo J, Balana-Fouce R (2008). Butyric acid based feed additives help protect broiler chickens from *Salmonella enteritidis* infection. Poult. Sci.

[11] Candela M, Maccaferri S, Turroni S, Carnevali P, Brigidi P (2010). Functional intestinal microbiome, new frontiers in prebiotic design. Int. J. Food Microbiol.

[12] Elmenawey M.A, Gharib H.B (2013). Effects of monospecies and multispecies probiotics on productive performance, intestinal histomorphological parameters and immune response in broilers. Egypt. J. Anim. Prod.

[13] NRC (1994). Nutrient Requirements of Poultry.

[14] Timbermont L, Lanckriet A, Gholamiandehkordi A, Pasmans F, Martel A, Haesebrouck F, Ducatelle R, Van Immerseel F (2009). Origin of *Clostridium perfringens* isolates determines the ability to induce necrotic enteritis in broilers. Comp. Immunol. Microbiol. Infect. Dis.

[15] North M.O (1984). Broiler, roaster, and capon management. Commercial Chicken Production Manual.

[16] Timmerman H, Veldman A, van den Elsen E, Rombouts F, Beynen A (2006). Mortality and growth performance of broilers given drinking water supplemented with chicken-specific probiotics. Poult. Sci.

[17] Swayne D.E, Glisson J.R, Jackwood M.W, Pearson J.E, Reed W.M (1998). A Laboratory Manual for the Isolation and Identification of Avian Pathogens.

[18] Gibb R.K, Taylor D, Wan T, Oconnor D, Doering D, Gercel-Taylor C (1997). Apoptosis as a measure of chemosensitivity to cisplatin and taxol therapy in ovarian cancer cell lines. Gynecol. Oncol.

[19] Ali F.K, El-Shafai S.A, Samhan F.A, Khalil W.K.B (2008). Effect of water pollution on expression of immune response genes of *Solea aegyptiaca* in Lake Qarun. Afr. J. Biotechnol.

[20] Elmegeed G, Khalil W, Mohareb R, Ahmed H, Abd-Elhalim M, Elsayed G (2011). Cytotoxicity and gene expression profiles of novel synthesized steroid derivatives as chemotherapeutic anti-breast cancer agents. Bioorgan. Med. Chem.

[21] Kulkarni R, Parreira V, Sharif S, Prescott J (2007). Immunization of Broiler chickens against *Clostridium perfringens* - Induced necrotic enteritis. Clin. Vac. Immunol.

[22] Hwang H.S, Han K.J, Ryu Y.H, Yang E.J, Kim Y.S, Jeong S.Y, Lee Y.S, Lee M.S, Koo S.T, Choi S.M (2009). Protective effects of electroacupuncture on acetylsalicylic acid-induced acute gastritis in chicken. World J. Gastroenterol.

[23] Houshmand M, Azhar K, Zulkifli I, Bejo M, Kamyab A (2011). Effects of nonantibiotic feed additives on performance, nutrient retention, gut pH, and intestinal morphology of broilers fed different levels of energy. J. Appl. Poult. Res.

[24] Bio-Rad Laboratories, Inc (2006). Real-Time PCR Applications Guide. Bulletin 5279.

[25] SAS Institute Inc (2004). SAS/STAT®9.1 User’s Guide.

[26] Duncan D.B (1955). Multiple range and multiple F testes. Biometrics.

[27] Vanhoutvin S.A, Troost F.J, Hamer H.M, Lindsey P.J, Koek G.H, Jonkers D.M, Kodde A, Venema K, Brummer R.J (2009). Butyrate-induced transcriptional changes in human colonic mucosa. PLoS One.

[28] Zhang W.H, Gao F, Zhu Q.F, Li C, Jiang Y, Dai S.F, Zhou G.H (2011). Dietary sodium butyrate alleviates the oxidative stress induced by corticosterone exposure and improves meat quality in broiler chickens. Poult. Sci.

[29] Gauthier R (2002). Intestinal health, the key to productivity (The case of organic acids). Scientific precongress Avicola IASA.

[30] Awaad M.H.H, Atta A.M, Elmenawey M, Shalaby B, Abdelaleem G.A, Madian K, Ahmed K, Marzin D, Benzoni G, Iskander D.K (2011). Effect of acidifiers on gastrointestinal tract integrity, zootechnical performance and colonization of *Clostridium perfringens* and aerobic bacteria in broiler chickens. J. Am. Sci.

[31] Jankowskia J, Juś kiewiczb J, Lichtorowicza K, Zdunċ zykb Z (2012). Effects of the dietary level and source of sodium on growth performance, gastrointestinal digestion and meat characteristics in turkeys. Anim. Feed Sci. Technol.

